# Adverse impact of tumor deposits in lymph node negative rectal cancer — a national cohort study

**DOI:** 10.1007/s00384-023-04365-1

**Published:** 2023-03-10

**Authors:** Simon Lundström, Erik Agger, Marie-Louise Lydrup, Fredrik Jörgren, Pamela Buchwald

**Affiliations:** 1 Department of Surgery, Skåne University Hospital, Lund University, Malmö, Sweden; 2 Department of Surgery, Helsingborg Hospital, Lund University, Helsingborg, Sweden

**Keywords:** Rectal cancer, Tumor deposits, N1c, Lymph node negative rectal cancer

## Abstract

**Purpose:**

This study aimed to investigate the prognostic effect of tumor deposits (TDs) in lymph node negative rectal cancer.

**Methods:**

Patients who had undergone surgery for rectal cancer with curative intention between 2011 and 2014 were extracted from the Swedish Colorectal Cancer Registry. Patients with positive lymph nodes, undisclosed TD status, stage IV disease, non-radical resections, or any outcome (local recurrence (LR), distant metastasis (DM) or mortality) within 90 days after surgery were excluded. TDs status was based on histopathological reports. Cox-regression analyses were used to examine the prognostic impact of TDs on LR, DM, and overall survival (OS) in lymph node–negative rectal cancer.

**Results:**

A total of 5455 patients were assessed for inclusion of which 2667 patients were analyzed, with TDs present in 158 patients. TD-positive patients had a lower 5-year DM-free survival (72.8%, *p* < *0.0001*) and 5-year overall survival (75.9%, *p* = *0.016*), but not 5-year LR-free survival (97.6%) compared to TD-negative patients (90.2%, 83.1% and 95.6%, respectively). In multivariable regression analysis, TDs increased the risk of DM [HR 4.06, 95% CI 2.72–6.06, *p* < *0.001*] and reduced the OS [HR 1.83, 95% CI 1.35–2.48, *p* < *0.001*]. For LR, only univariable regression analysis was performed which showed no increased risk of LR [HR 1.88, 95% CI 0.86–4.11, *p* = *0.11*].

**Conclusion:**

TDs are a negative predictor of DM and OS in lymph node–negative rectal cancer and could be taken into consideration when planning adjuvant treatment.

**Supplementary Information:**

The online version contains supplementary material available at 10.1007/s00384-023-04365-1.

## Background

Tumor deposits (TDs) have been given increased importance as a prognostic factor in colorectal cancer (CRC). According to the eighth edition of the American Joint Committee on Cancer (AJCC) TNM staging manual, TDs are defined as discrete tumor nodules found within the lymph drainage area of a primary CRC containing no identifiable lymphatic, vascular, or neural structures [1]. Correlations between TDs and increased risk of distant metastasis (DM) and impaired survival have been demonstrated in previous CRC cohorts [2]. Recent advances have indicated that colon and rectal cancer differ regarding molecular genetics and metastatic pattern [3]. As colon cancer often is overrepresented in CRC cohorts, further evaluation of TDs as a risk factor in rectal cancer is of interest.

TDs without any positive lymph nodes are since the seventh TNM edition classified as N1c, corresponding to stage III cancer which justifies adjuvant treatment according to national guidelines [4, 5]. Histological variables such as vascular/lymphatic (V/L) infiltration, perineural growth, or high-grade tumor have been shown to be important additions to the TNM classification for predicting the risk of DM and survival in rectal cancer patients [2, 6]. Furthermore, variables such as TDs, tumor budding, mismatch repair deficiency, and KRAS status may aid in predicting oncological outcomes for example DM and overall survival (OS) [7–9]. In a recent rectal cancer study, the presence of TDs was associated with an increased risk of local recurrence (LR), DM, and decreased OS [10].

TD-positive rectal cancer is associated with synchronous regional lymph node metastasis and DM thereby potentially resulting in a more advanced stage [2]. A negative prognostic effect of TDs in lymph node–negative rectal cancer has been suggested, but the results have either been extrapolated from mixed CRC cohorts or from small rectal cancer studies and require further validation as an independent prognostic factor [11, 12].

The aim of this study was to evaluate if TDs are associated with increased risk of LR, DM, and decreased OS in patients with lymph node–negative rectal cancer. We hypothesized that TDs have a negative impact on LR, DM, and OS in lymph node–negative rectal cancer.

## Method

### Study population

Data from the Swedish Colorectal Cancer Registry (SCRCR) was extracted for patients that underwent abdominal surgery (Hartmann’s procedure, anterior resection, or abdominoperineal resection) with a curative intention for rectal cancer between January 1st, 2011, and December 31st, 2014. SCRCR is a national database which includes 98.8% of rectal cancer patients and has a variable agreement of 90% [13].

Patients with undefined TD-status, lymph node–positive rectal cancer (pN1a-b or pN2a-b), stage IV disease, or non-radical resections were excluded. Patients with study outcomes (LR, DM, or mortality) within 90 days after surgery were excluded as this may represent cases of post-operative mortality, non-recognized non-radical surgical resections, or undiagnosed metastatic spread at the time of surgery. After exclusion, patients were divided into two groups: TD-positive and TD-negative. All included patients were lymph node negative according to histopathological reports. Recurrence data was collected continuously and in conjunction with standardized follow-up at 3 and 5 years. Survival data was collected from the Swedish Cause of Death Register on September 2nd, 2020.

Neoadjuvant treatment was given based on the clinical stage following a multidisciplinary team conferences according to the clinical routines and could be either a short course radiotherapy, a long course of radiotherapy in combination with capecitabine, or in rare cases systemic chemotherapy only.

### Statistical analysis

Categorical variables were presented as frequency (%) and group differences were investigated using the Pearson chi-square test with post hoc Bonferroni correction when appropriate. Continuous variables were presented as median [interquartile range] with differences examined using the Kruskal–Wallis test. The relation of TDs and oncological outcomes (LR, DM, and OS) was assessed using uni- and multivariable cox-regression analyses.

Patients with unspecified follow-up status due to missing data were censored for LR and DM for cox-regression and Kaplan–Meier analysis at day zero. Patients with unspecified vital status due to emigration were censored for OS in cox-regression and Kaplan–Meier analysis at the day of emigration.

Confounding variables were identified prior to data analysis with the aid of directed acyclic graphs for multivariable analysis and limited by the numbers of outcomes. For DM, multivariable analysis was adjusted for age, sex, neoadjuvant treatment (radiotherapy, chemotherapy, or both), clinical staging, pathological staging, and adjuvant treatment (radiotherapy, chemotherapy, or both). For OS, the previously mentioned variables were used with the addition of the American Society of Anesthesiologists (ASA) classification. For both DM and OS, the chosen variables were to some extent proxy for V/L infiltration, perineural growth, high-grade tumor, and tumor height. For multivariable analysis, patients with rectal cancer with histopathological stage 0 were excluded, and stage I was set as the reference stage.

Kaplan–Meier curves were used to determine the 5-year cumulative LR and DM as well as the 5-year cumulative OS with differences investigated using the log-rank test. Statistical analyses were performed using IBM^®^ SPSS^®^ Statistics version 28.0 for Windows^®^ (IBM Crop, Armonk, NY, USA) and R version 4.1.2 (R Core Team 2022, Vienna, Austria). All figures were created in R. A *p*-value < 0.05 was considered statistically significant.

### Ethical considerations

This study was approved by the Swedish Ethical Review Authority (DNR: 2020–01769). Registration in SCRCR is voluntary with the possibility to delete personal health data at any time. Registration in the Cause of Death Register is mandatory for all citizens by law and does not require any consent.

### Definitions

*Rectal cancer* was defined as adenocarcinoma with the lower border located within 15 cm from the anal verge measured with rigid sigmoidoscopy.

*TDs* were defined according to the seventh edition of the TNM staging system as a foci of tumor cells discontinuous from the primary tumor located within the lymph drainage of the perirectal fat tissue without signs of lymphatic tissue [5]. All histopathological specimens have been examined by a pathologist within the clinical routine.

*N1c* was defined as TD-positive lymph node–negative rectal cancer.

*Non-radical resection* was defined as R2, 0.0 mm or unspecified circumferential resection margin, or 0.0 mm or unspecified distal resection margin.

*LR* was defined as the recurrence of local extraperitoneal tumor, lymph node tumor growth, intraluminal tumor recurrence, or peritoneal tumor growth below the promontory.

*DM* was defined as the recurrence of a tumor in any organ outside the small pelvis.

*OS* was defined as the duration between the date of surgery and death of any cause.

## Results

### Study population

**Fig. 1:**
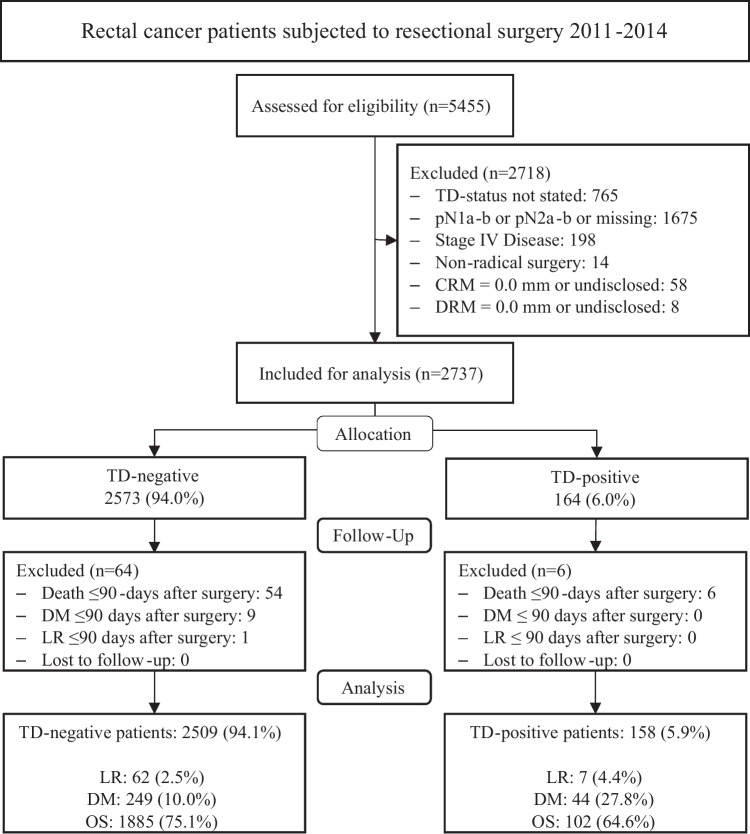
Study flow chart. TD, tumor deposit; CRM, circumferential resection margin; DRM, distal resection margin; LR, local recurrence; DM, distant metastasis; OS, overall survival. LR, DM, and OS are presented as *n* (frequency) at the last follow-up for all participants

The study flowchart is presented in Fig. 1. Between January 2011 and December 2014, a total of 5455 patients from the SCRCR with rectal adenocarcinoma fulfilled the inclusion criteria and were assessed for eligibility. Two thousand seven hundred eighteen patients were excluded in accordance with the predetermined exclusion criteria. Of the remaining 2737 patients, 6 TD-positive and 64 TD-negative patients had an early (< 90 days) event of LR, DM, or death following surgery and were excluded. Thus, 158 TD-positive and 2509 TD-negative patients comprised the final study cohort.

Baseline group characteristics are presented in Table 1. TD-positive patients demonstrated a higher clinical staging and more frequently received neoadjuvant therapy. Moreover, TD-positive patients more often exhibited V/L infiltration, perineural growth, and high-grade tumor and received adjuvant therapy to a higher extent. At histopathological evaluation, 5% of the TD-negative patients exhibited complete tumor response after neoadjuvant treatment. The median follow-up time was 60 [IQR 55–63] months for TD-positive patients and 60 [IQR 58–63] months for TD-negative patients.

**Table 1:** Group characteristics for TD + and TD − patients with node negative rectal cancer

		TD-negative	TD-positive
**Patients (*n*)**		2509	158
**Sex (*n*)**	Male	1498 (59.7)	91 (57.6)
	Female	1011 (40.3)	67 (42.4)
**Age**	Years	69 [62–76]	69 [61–77]
**BMI**	kg/m2	25 [23–28]	25 [23–27]
**ASA-score**	ASA 1–2	1929 (76.9)	115 (72.8)
	ASA 3–4	569 (22.7)	41 (25.9)
	Missing	11 (0.4)	2 (1.3)
**Tumor height**	Low (0–5 cm)	767 (30.6)	40 (25.3)
	Medium (6–10 cm)	1024 (40.8)	67 (42.4)
	High (11–15 cm)	690 (27.5)	49 (31.0)
	Missing	28 (1.1)	2 (1.3)
**Clinical stage**	I	658 (26.2)	21 (13.3)
	II	672 (26.8)	33 (20.9)
	III	1133 (45.2)	104 (65.8)
	Missing	46 (1.8)	0 (0.0)
**cT-stage**	cT1–2	780 (31.1)	20 (12.7)
	cT3	1222 (48.7)	99 (62.7)
	cT4	404 (16.1)	35 (22.2)
	Missing	103 (4.1)	4 (2.5)
**cN-stage**	cN0	1215 (48.4)	45 (28.5)
	cN1–2	1133 (45.2)	104 (65.8)
	Missing	161 (6.4)	9 (5.7)
**Any neoadjuvant therapy**	RT/CRT/CHT	1662 (66.2)	128 (81.0)
	None	846 (33.7)	30 (19.0)
	Missing	1 (0.0)	0 (0.0)
**Any complication**	Yes	938 (37.4)	67 (42.4)
	No	1571 (62.6)	91 (57.6)
**Histopathologic stage**	0	126 (5.0)	0 (0.0)
	I	1206 (48.1)	0 (0.0)
	II	1177 (46.9)	0 (0.0)
	III	0 (0.0)	158 (100.0)
**pT-stage**	pT0	122 (4.9)	5 (3.2)
	pT1	284 (11.3)	5 (3.2)
	pT2	922 (36.7)	23 (14.6)
	pT3	1095 (43.6)	110 (69.6)
	pT4	82 (3.3)	15 (9.5)
	Missing	4 (0.2)	0 (0.0)
**CRM**	> 1.0 mm	2443 (97.4)	144 (91.1)
	1.0–0.1 mm	66 (2.6)	14 (8.9)
**Vascular/lymphatic infiltration**	Yes	247 (9.8)	57 (36.1)
	No	2243 (89.4)	101 (63.9)
	Missing	19 (0.8)	0 (0.0)
**Perineural growth**	Yes	166 (6.6)	42 (26.6)
	No	2256 (89.9)	111 (70.3)
	Missing	87 (3.5)	5 (3.2)
**High-grade tumor**	Yes	229 (9.1)	27 (17.1)
	No	2088 (83.2)	117 (74.1)
	Missing	192 (7.7)	14 (8.9)
**Adjuvant therapy**	Yes	244 (9.7)	61 (38.6)
	No	2258 (90.0)	97 (61.4)
	Missing	7 (0.3)	0 (0.0)
**Local recurrence**	Yes	62 (2.5)	7 (4.4)
	No	2439 (97.2)	151 (95.6)
	Missing	8 (0.3)	0 (0.0)
**Distant metastasis**	Yes	249 (9.9)	43 (27.2)
	No	2252 (89.8)	115 (72.8)
	Missing	8 (0.3)	0 (0.0)
**Mortality**	Deceased	615 (24.5)	55 (34.8)
	Alive at follow-up	1885 (75.1)	102 (64.6)
	Emigrated	9 (0.4)	1 (0.6)
**Follow-up (median)**	Months	60 [58–63]	60 [55–63]

Continuous values are presented as median [interquartile range]. Categorical values are presented as frequency (%)

### Local recurrence, distant metastasis, and overall survival

TD-positive patients experienced more DM (27.2% vs 9.9%, *p* =  < *0.001*) and decreased survival (64.6%, vs 75.1%, *p* = *0.012*), but not LR (4.4% vs 2.5%, *p* = *0.135*) compared to TD-negative patients (Table 1). Univariable cox-regression analysis showed a significantly increased rate of DM (HR 3.12, 95% CI 2.26–4.31, *p* < *0.001*) and decreased OS (HR 1.58, 95% CI 1.20–2.08, *p* = *0.001*) for TD-positive patients compared to TD-negative patients, but no difference in recurrence rate for LR (HR 1.88, 95% CI 0.86–4.11, *p* = *0.11*) (Table 2). Multivariable analysis was not performed for LR due to a low number of events. TDs remained a statistically significant factor in multivariable analysis for DM (HR 4.06, 95% CI 2.72–6.06, *p* < *0.001*) and decreased OS (HR 1.83, 95% CI 1.35–2.48, *p* < *0.001*) (Table 2).

**Table 2:** Hazard ratio of local recurrence, distant metastasis, and overall survival for TD-positive lymph node negative rectal cancer

	**Local recurrence**			**Distant metastasis**			**Overall survival**		
	**Univariable**			**Univariable**			**Univariable**		
	*n*	HR (CI 95%)	*p*-value	*n*	HR (CI 95%)	*p*-value	n	HR (CI 95%)	*p*-value
TD-negative	2501	1	Ref	2509	1	Ref	2509	1	Ref
TD-positive	158	1.88 (0.86–4.11)	0.114	158	3.12 (2.26–4.31)	< 0.001	158	1.58 (1.20–2.08)	0.001
				**Multivariable**^**a**^			**Multivariable**^**b**^		
				*n*	HR (CI 95%)	*p*-value	*n*	HR (CI 95%)	*p*-value
TD-negative				2325	1	Ref	2320	1	Ref
TD-positive				158	4.06 (2.72–6.06)	< 0.001	156	1.83 (1.35–2.48)	< 0.001

Cox-regression analysis of local recurrence, distant metastasis and overall survival at the end of follow-up

^a^Multivariable analysis adjusted for age, sex, neoadjuvant therapy, cTNM-stage, pTNM-stage, and adjuvant therapy

^b^Multivariable analysis adjusted for age, sex, ASA classification, neoadjuvant therapy, cTNM-stage, pTNM-stage, and adjuvant therapy. For the pTNM stage, stage 0 was excluded from the multivariable analysis

**Fig. 2:**
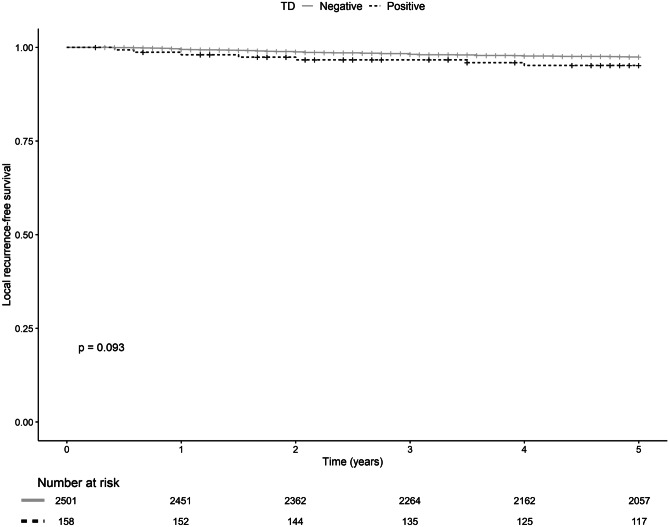
5-year local recurrence-free survival for TD-positive (straight gray line) and TD-negative (dotted black line) with censored patients marked with a vertical line and corresponding tables of number at risk

**Fig. 3:**
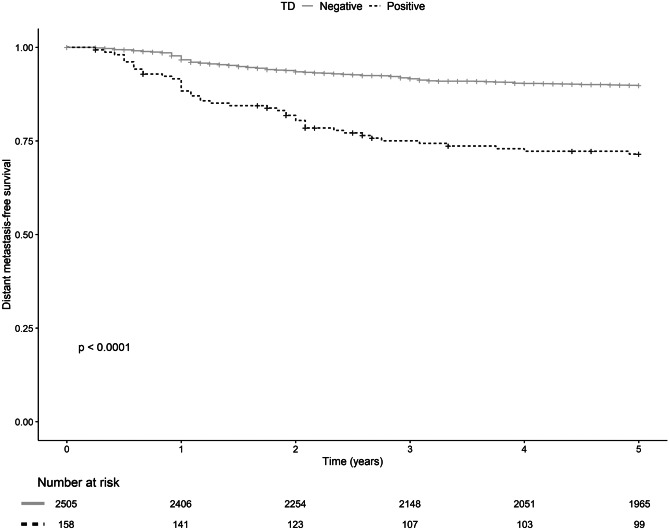
5-year distant metastasis free–survival for TD-positive (straight gray line) and TD-negative (dotted black line) with censored patients marked with a vertical line and corresponding tables of number at risk

**Fig. 4:**
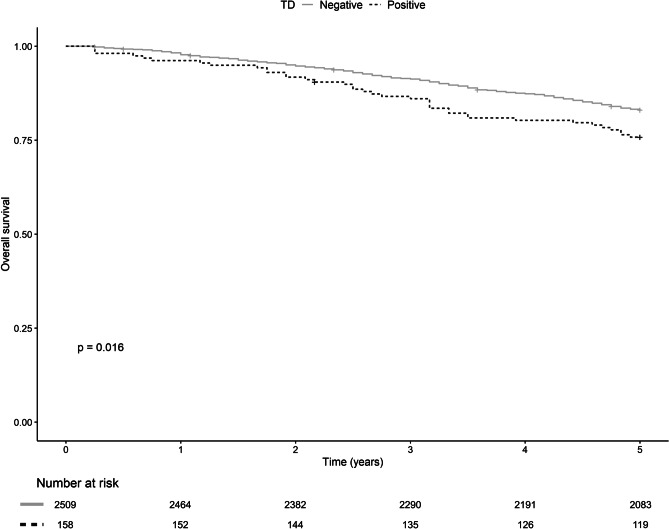
5-year overall survival for TD-positive (straight gray line) and TD-negative (dotted black line) with censored patients marked with a vertical line and corresponding tables of number at risk

TD-positive patients had a lower 5-year DM-free survival (72.8%, *p* < *0.0001*, Fig. 2) and had a decreased 5-year overall survival rate (75.9%, *p* = *0.016*, Fig. 3) compared to TD-negative patients (90.2% and 83.1%, respectively). No significant difference in 5-year LR-free survival was seen (Fig. 4). Furthermore, TDs exhibit a negative additive effect on 5-year OS together with histopathological risk factors (V/L infiltration, perineural growth, or high-grade tumor) (Supplement Fig. 5). However, TD individual effect on OS was not higher than the individual effect of V/L infiltration, perineural growth, or high-grade tumor (Supplement Fig. 6).

### Examined lymph nodes

In the cohort, 470 patients (17.6%) had < 12 histologically examined negative lymph nodes. Compared to patients with ≥ 12 examined lymph nodes, those with < 12 examined lymph nodes had a lower ASA classification, more frequently underwent neoadjuvant therapy, and were more often stage 0 or stage I at the histopathological examination of the rectal specimen following surgery. There were no significant differences in the prevalence of TDs or any of the examined outcomes between these groups (Supplement Table 3).

## Discussion

The present study showed that TDs are a negative prognostic factor for DM and OS in lymph node–negative rectal cancer regardless of the number of examined lymph nodes using population-based data, while no prognostic effect on LR could be shown. This study also confirms the clinical feasibility of the current N1c stage for lymph node–negative rectal cancer. TDs might have a possible additive prognostic effect on V/L infiltration, perineural growth, and high-grade tumor.

The importance of TDs in CRC has been demonstrated [2, 10–12, 14–17]. However, few similar analyses have been performed in lymph node–negative rectal cancer cohorts. Agger et al. showed that TDs are a negative prognostic factor for rectal cancer similar to N1a-b [10]. Wei et al. indicated that TDs were a significant independent prognostic risk factor in lymph node–negative rectal cancer who had undergone preoperative radiotherapy [15]. In the current study of lymph node–negative rectal cancer, TD-positive patients had a 5-year DM-free survival of 73% and nearly four times higher risk of DM compared to TD-negative patients, independently of preoperative radiotherapy status. This is comparable to N1a-b, which has a 5-year DM-free survival of 74% [10].

TDs has been shown to be associated with an increased risk of LR in rectal cancer but this has not been independently proven in lymph node–negative rectal cancer [10, 18]. The current study could not find a significant difference in LR incidence between TD-negative and TD-positive patients. This may be due to a few registered events which also precluded multivariable analysis. A larger group with more events is necessary to rule out a significant difference.

The current study did not investigate the prognostic impact of TDs on lymph node–positive rectal cancer. As both the seventh and eighth edition of the TNM cancer staging manual only takes TDs into account in lymph node–negative rectal cancer (N1c), the staging of lymph node–positive rectal cancer remains unaltered independently of the presence or numbers of TDs. For CRC, Pricolo et al. showed a negative prognostic impact of multiple TDs in lymph node–negative colon cancer whereas Pu et al. demonstrated a negative prognostic impact of TDs also in lymph node–positive CRC [17, 19]. Consequently, requests have been made for modifications of the current TNM N-staging to account for the increased risk of multiple TDs in CRC [14, 17, 19, 20]. If comparable results were to be presented for rectal cancer specifically, further support would exist for a novel N-staging method.

TDs were associated with higher clinical staging, more frequent use of neoadjuvant therapy, and a higher rate of synchronous V/L infiltration, perineural growth, and high-grade tumor in lymph node–negative cancer compared to TD-negative patients, which is in accordance with previous research [14]. As demonstrated in Fig. 6, Appendix, TDs do not seem to have a greater prognostic effect by themselves compared to isolated V/L infiltration, perineural growth, or high-grade tumor. However, TDs appear to have an additive effect when present simultaneously with V/L infiltration, perineural growth, and/or high-grade tumor, especially for patients with total synchronous presence of risk factors (Fig. 5, Appendix). Unfortunately, these groups had few patients and a more extensive database is required to draw more definitive conclusions.

## Limitations

The data used was collected between 2011 and 2014 and histopathological results were based on the seventh edition of TNM. In the eighth edition, which was implemented clinically in 2016, emphasis was put on nodal origin. Differentiation was made based on residual lymphatic, vascular, or neural tissue, and only in the absence of these findings, a nodule was classified as a TD [1]. However, the prognostic importance of this classification has not been demonstrated. A study exploring the interobserver agreement for detecting TDs between pathologists in colon cancer according to the seventh TNM edition showed a complete agreement of 44% in a selected cohort of histologically challenging specimen [21]. For the eighth edition, a complete agreement of 6% and a general agreement of 52% were shown in unfiltered cases from the daily clinical practice [22]. In the same study, the usage of a nodal versus non-nodal classification instead resulted in a complete agreement of 40% and an overall agreement of 73.5% [22]. The low agreements for the seventh and eighth TNM editions have been attributed to a lack of objective criteria and calls have been made for more objective and simplified classifications [22]. These findings are likely applicable to rectal cancer as well, and the utilization of the seventh edition TNM in the present study should not have caused a significant impact on the results.

Out of the original 5455 patients assessed for eligibility, 765 (14%) patients had unknown TD status. Of these, 372 fulfilled additional exclusion criteria. The data for the remaining 392 (7%) patients is presented in supplement Table 4. Patients with unknown TD status were missing it at random but may also represent more difficult specimens where histological characteristics cannot be determined. Classification of tumor grade was more often missing compared to those with known TD status (15.8% vs 7.7%). Patients with unknown TD status also had higher mortality compared to those with known TD status (30.5% vs 25.1%). Whether this is a type 1 error or a true difference remains unclear.

Distant metastasis had few outcomes (*n* = 44) and did not fulfill the rule of ten for the six confounding factors that were adjusted for. Vittinghoff et al. showed the rule of ten to be too constricted and that 5–9 events per adjusted variable often resulted in similar error rates as 10–16 events per adjusted variable [23]. The use of six adjusting variables for 44 events (i.e., 7.3 events per variable) should have little effect on the results.

TNM guidelines state that < 12 examined lymph nodes count as a negative prognostic factor [1], which was the case for 17.6% of our study population. Neoadjuvant treatment aims to downstage tumors and leads to a complete response in as high as 20% of cases and is known to reduce the lymph node yield [24, 25]. Whether < 12 examined lymph nodes remain a negative prognostic factor after neoadjuvant treatment requires further investigation [26, 27]. As there were no significant differences in the prevalence of TDs or any of the examined outcomes in the current study, patients with < 12 examined lymph nodes were included.

The presence of TDs after neoadjuvant treatment appears to be a poor prognostic marker and may be a sign of tumor resilience or a case of tumor fragmentation, which is a negatively associated type of tumor regression [9]. If neoadjuvant treatment affects the number of TDs found and whether the inclusion of TDs in preclinical staging using radiological examinations would add additional information remain to be studied.

## Conclusions

This study shows that TDs are a strong independent negative predictor for DM and OS in lymph node–negative rectal cancer. Thus, TDs could be taken into consideration when considering adjuvant treatment in rectal cancer patients. More efforts should be done to identify TDs preoperatively.

### Supplementary Information

Below is the link to the electronic supplementary material.

Supplementary file1 (DOCX 108 KB)

## Data Availability

Data could be made available upon reasonable request to the authors.
