# Supplementary material for: Adverse impact of tumor deposits in lymph node negative rectal cancer — a national cohort study
Source: Int J Colorectal Dis. 2023 Mar 10;38(1):66. doi: 10.1007/s00384-023-04365-1 (PMC10006276; doi:10.1007/s00384-023-04365-1)
Supplement: Supplementary file 1 — Supplementary file1 (DOCX 108 KB) [file 384_2023_4365_MOESM1_ESM.docx]

# **Appendix**:

**Suppl. Fig. 5**


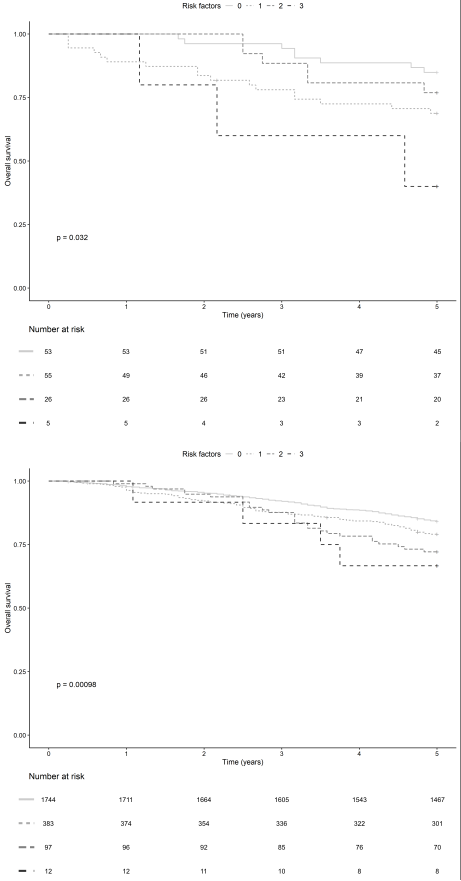


**Additive effect of tumor deposits on overall survival to classic histological risk factors (vascular/lymphatic infiltration, perineural growth or high-grade tumor).** 5-year overall survival for TD-positive patients (**top**) and 5-year survival for TD-negative patients (**bottom**) with 0 (straight light gray line), 1 (dotted light gray line), 2 (dashed dark gray line) or 3 (dashed black line) risk factors, with corresponding tables of number at risk

**Suppl. Fig. 6**

**
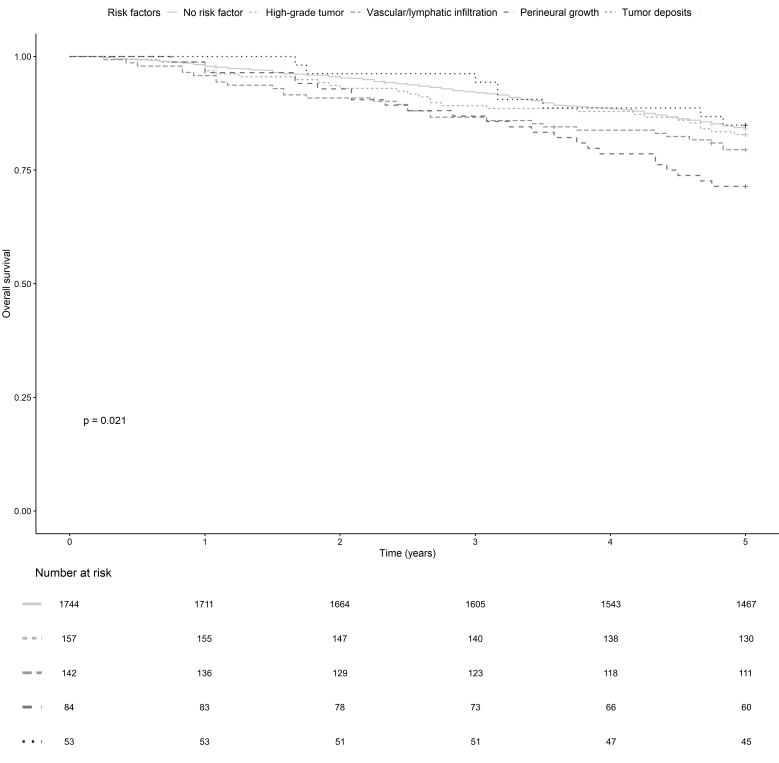
**

**Effects of tumor deposits on overall survival compared to no risk factors or classic histological risk factors (vascular/lymphatic infiltration, perineural growth or high-grade tumor).** 5-year overall survival for patients with an isolated risk factor; TD (dotted black line), vascular/lymphatic infiltration (dashed light grey line), perineural growth (dashed black line), high-grade tumor (dotted light grey line), or no risk factor (straight light grey line), with corresponding table of number at risk

**Suppl. Table 3.** **Group characteristics for patients with ≥ 12 and < 12 examined lymph nodes**

|  |  | ≥ 12 examined lymph nodes | < 12 examined lymph nodes |
| --- | --- | --- | --- |
| **Patients (n)** |  | 2197 | 470 |
| **Sex (n)** | Male | 1319 (60.0) | 270 (57.4) |
|  | Female | 878 (40.0) | 200 (42.6) |
| **Age (median)** | Years | 69 [61-76] | 70 [64-76] |
| **BMI (median)** | kg/m2 | 25 [23-28] | 26 [23-28] |
| **ASA-score** | ASA 1 | 1686 (76.7) | 358 (76.2) |
|  | ASA 4 | 498 (22.7) | 112 (23.8) |
|  | Missing | 13 (0.6) | 0 (0.0) |
| **Tumor height** | Low (0-5 cm) | 591 (26.9) | 216 (46.0) |
|  | Medium (6-10 cm) | 933 (42.5) | 158 (33.6) |
|  | High (11-15 cm) | 647 (29.4) | 92 (19.6) |
|  | Missing | 26 (1.2) | 4 (0.9) |
| **Clinical stage** | I | 545 (24.8) | 134 (28.5) |
|  | II | 587 (26.7) | 118 (25.1) |
|  | III | 1027 (46.7) | 210 (44.7) |
|  | Missing | 38 (1.7) | 8 (1.7) |
| **cT-stage** | cT1-2 | 639 (29.1) | 161 (34.3) |
|  | cT3 | 1123 (51.1) | 198 (42.1) |
|  | cT4 | 351 (16.0) | 88 (18.7) |
|  | Missing | 84 (3.8) | 23 (4.9) |
| **cN-stage** | cN0 | 1025 (46.7) | 235 (50.0) |
|  | cN1-2 | 1027 (46.7) | 210 (44.7) |
|  | Missing | 145 (6.6) | 25 (5.3) |
| **Any neoadjuvant therapy** | RT/CRT/CHT | 1433 (65.2) | 357 (76.0) |
|  | None | 763 (34.7) | 113 (24.0) |
|  | Missing | 1 (0.0) | 0 (0.0) |
| **Any surgical complication** | Yes | 808 (36.8) | 197 (41.9) |
|  | No | 1389 (63.2) | 273 (58.1) |
| **Histopathologic stage** | 0 | 84 (3.8) | 42 (8.9) |
|  | I | 970 (44.2) | 236 (50.2) |
|  | II | 1013 (46.1) | 164 (34.9) |
|  | III | 130 (5.9) | 28 (6.0) |
| **pT-stage** | pT0 | 83 (3.8) | 44 (9.4) |
|  | pT1 | 216 (9.8) | 73 (15.5) |
|  | pT2 | 777 (35.4) | 168 (35.7) |
|  | pT3 | 1035 (47.1) | 170 (36.2) |
|  | pT4 | 83 (3.8) | 14 (3.0) |
|  | Missing | 3 (0.1) | 1 (0.2) |
| **pN-stage** | pN0 | 2067 (94.1) | 442 (94.0) |
|  | pN1c | 130 (5.9) | 28 (6.0) |
| **CRM** | >1.0 mm | 2135 (97.2) | 452 (96.2) |
|  | 1.0-0.1 mm | 62 (2.8) | 18 (3.8) |
| **TD** | Positive | 130 (5.9) | 28 (6.0) |
|  | Negative | 2067 (94.1) | 442 (94.0) |
| **Vascular/lymphatic infiltration** | Yes | 259 (11.8) | 45 (9.6) |
|  | No | 1922 (87.5) | 422 (89.8) |
|  | Missing | 16 (0.7) | 3 (0.6) |
| **Perineural growth** | Yes | 169 (7.7) | 39 (8.3) |
|  | No | 1950 (88.8) | 417 (88.7) |
|  | Missing | 78 (3.6) | 14 (3.0) |
| **High-grade tumor** | Yes | 203 (9.2) | 53 (11.3) |
|  | No | 1848 (84.1) | 357 (76.0) |
|  | Missing | 146 (6.6) | 60 (12.8) |
| **Adjuvant therapy** | Yes | 234 (10.7) | 71 (15.1) |
|  | No | 1958 (89.1) | 397 (84.5) |
|  | Missing | 5 (0.2) | 2 (0.4) |
| **Local recurrence** | Yes | 54 (2.5) | 15 (3.2) |
|  | No | 2137 (97.3) | 453 (96.4) |
|  | Missing | 6 (0.3) | 2 (0.4) |
| **Distant metastasis** | Yes | 230 (10.5) | 62 (13.2) |
|  | No | 1961 (89.3) | 406 (86.4) |
|  | Missing | 6 (0.3) | 2 (0.4) |
| **Mortality** | Deceased | 534 (24.3) | 136 (28.9) |
|  | Alive at follow-up | 1654 (75.3) | 333 (70.9) |
|  | Emigrated | 9 (0.4) | 1 (0.2) |
| **Follow-up (median)** | Months | 60 [58-63] | 60 [57-62] |

Continuous values are presented as median [interquartile range]. Categorical values are presented as frequency (%).

**Suppl. Table 4.** **Group characteristics for patients with known and unknown TD-status**

|  |  | Known TD-status | Unknown TD-status |
| --- | --- | --- | --- |
| **Patients (n)** |  | 2667 | 393 |
| **Sex (n)** | Male | 1589 (59.6) | 243 (61.8) |
|  | Female | 1078 (40.4) | 150 (38.2) |
| **Age (median)** | Years | 69 [62-76] | 69 [62-77] |
| **BMI (median)** | kg/m2 | 25 [23-28] | 26 [23-28] |
| **ASA-score** | ASA 1-2 | 2044 (76.6) | 311 (79.1) |
|  | ASA 3-4 | 610 (22.9) | 79 (20.1) |
|  | Missing | 13 (0.5) | 3 (0.8) |
| **Tumor height** | Low (0-5 cm) | 807 (30.3) | 122 (31.0) |
|  | Medium (6-10 cm) | 1091 (40.9) | 153 (38.9) |
|  | High (11-15 cm) | 739 (27.7) | 114 (29.0) |
|  | Missing | 30 (1.1) | 4 (1.0) |
| **Clinical stage** | I | 679 (25.5) | 103 (26.2) |
|  | II | 705 (26.4) | 106 (27.0) |
|  | III | 1237 (46.4) | 170 (43.3) |
|  | Missing | 46 (1.7) | 14 (3.6) |
| **cT-stage** | cT1-2 | 800 (30.0) | 108 (27.5) |
|  | cT3 | 1321 (49.5) | 212 (53.9) |
|  | cT4 | 439 (16.5) | 43 (10.9) |
|  | Missing | 107 (4.0) | 30 (7.6) |
| **cN-stage** | cN0 | 1260 (47.2) | 186 (47.3) |
|  | cN1-2 | 1237 (46.4) | 170 (43.3) |
|  | Missing | 170 (6.4) | 37 (9.4) |
| **Any neoadjuvant therapy** | RT/CRT/CHT | 1790 (67.1) | 237 (60.3) |
|  | None | 876 (32.8) | 156 (39.7) |
|  | Missing | 1 (0.0) | 0 (0.0) |
| **Any surgical complication** | Yes | 1005 (37.7) | 159 (40.5) |
|  | No | 1662 (62.3) | 234 (59.5) |
| **Histopathologic stage** | 0 | 126 (4.7) | 16 (4.1) |
|  | I | 1206 (45.2) | 170 (43.3) |
|  | II | 1177 (44.1) | 207 (52.7) |
|  | III | 158 (5.9) | 0 (0.0) |
| **pT-stage** | pT0 | 127 (4.8) | 13 (3.3) |
|  | pT1 | 289 (10.8) | 29 (7.4) |
|  | pT2 | 945 (35.4) | 141 (35.9) |
|  | pT3 | 1205 (45.2) | 192 (48.9) |
|  | pT4 | 97 (3.6) | 15 (3.8) |
|  | Missing | 4 (0.1) | 3 (0.8) |
| **CRM** | >1.0 mm | 2587 (97.0) | 380 (96.7) |
|  | 1.0-0.1 mm | 80 (3.0) | 13 (3.3) |
| **Vascular/lymphatic infiltration** | Yes | 304 (11.4) | 43 (10.9) |
|  | No | 2344 (87.9) | 314 (79.9) |
|  | Missing | 19 (0.7) | 36 (9.2) |
| **Perineural growth** | Yes | 208 (7.8) | 30 (7.6) |
|  | No | 2367 (88.8) | 303 (77.1) |
|  | Missing | 92 (3.4) | 60 (15.3) |
| **High-grade tumor** | Yes | 256 (9.6) | 35 (8.9) |
|  | No | 2205 (82.7) | 296 (75.3) |
|  | Missing | 206 (7.7) | 62 (15.8) |
| **Adjuvant therapy** | Yes | 309 (11.6) | 34 (8.7) |
|  | No | 2351 (88.2) | 359 (91.3) |
|  | Missing | 7 (0.3) | 0 (0.0) |
| **Local recurrence** | Yes | 69 (2.6) | 12 (3.1) |
|  | No | 2590 (97.1) | 381 (96.9) |
|  | Missing | 8 (0.3) | 0 (0.0) |
| **Distant metastasis** | Yes | 292 (10.9) | 52 (13.2) |
|  | No | 2367 (88.8) | 341 (86.8) |
|  | Missing | 8 (0.3) | 0 (0.0) |
| **Mortality** | Deceased | 670 (25.1) | 120 (30.5) |
|  | Alive at follow-up | 1987 (74.5) | 273 (69.5) |
|  | Emigrated | 10 (0.4) | 0 (0.0) |
| **Follow-up (median)** | Months | 60 [58-63] | 60 [57-64] |

Continuous values are presented as median [interquartile range]. Categorical values are presented as frequency (%).
